# Deep-Eutectic-Solvent-Decorated Metal–Organic Framework for Food and Environmental Sample Preparation

**DOI:** 10.3390/foods13223614

**Published:** 2024-11-13

**Authors:** Wanlin Deng, Chen Fan, Ruixue Zhang, Ming Jin

**Affiliations:** School of Light Industry Science and Engineering, Beijing Technology and Business University, Beijing 100048, China

**Keywords:** deep eutectic solvent, sample preparation, metal-organic framework, green solvent, food safety

## Abstract

Deep eutectic solvent (DES) is distinguished by its unique solvent properties, chemical stability, and eco-friendly nature, which are pivotal in a spectrum of chemical processes. It enhances the sample preparation process by increasing efficiency and minimizing the environmental impact. Metal–organic frameworks (MOFs), which are porous structures formed through coordination bonds between metal ions and organic ligands, are defined by their adjustable pore dimensions, extensive surface areas, and customizable architectures. The integration of DES within MOF to create DES@MOF capitalizes on the beneficial attributes of both materials, augmenting MOFs’ stability and versatility while providing a multifunctional carrier for DES. This composite material is both highly stable and readily tunable, establishing it as a leading contender for applications in sample preparation for food and environmental samples. This comprehensive review explores the application of DES-decorated MOF in food and environmental sample preparation and highlights the expansive potential of DES@MOF in diverse fields. We provide a detailed analysis of the characteristics of DES@MOF and its individual components, methods for decorating MOFs with DES, the advantages of these composite materials in sample pretreatment, and their specific applications in food safety and environmental monitoring. DESs are employed to modify MOFs, offering a multitude of benefits that can substantially improve the overall performance and applicability of MOFs. The review also discusses current challenges and future directions in this field, offering valuable insights for further research and development. The synergistic effects of DES and MOFs offer new opportunities for applications in food safety and other areas, leading to the development of more efficient, sensitive, and environmentally friendly analytical methods. This collaboration paves the way for sustainable technologies and innovative solutions to complex challenges.

## 1. Introduction

For 2030, the United Nations’ Sustainable Development Goals prioritize ensuring food security and safety by advocating for production systems for sustainable food [1]. Food safety is a critical component of public health that ensures that the food we consume is free from harmful substances that could pose a risk to our health. The importance of food safety cannot be overstated, as it is directly linked to the prevention of foodborne diseases, which affect millions of people worldwide. Unsafe food may contain harmful bacteria, viruses, parasites, or chemical substances, which can cause over 200 diseases ranging from diarrhea to cancers [2]. The detection of these hazardous substances is therefore paramount to prevent illness and ensure the safety of the food supply. Sample preparation stands as an important phase in the realm of analytical chemistry; it is the linchpin that ensures the fidelity of experimental or analytical outcomes [3]. Proper preparation is essential to mitigate the presence of impurities that may complicate the sample matrix, as untreated samples risk rendering measurements, conclusions, or results significantly flawed [4,5]. Moreover, meticulous sample handling is crucial to prevent contamination, which has the potential to greatly distort results and, in turn, invalidate entire experimental endeavors, thereby obviating the investment of time and resources [6]. Traditional approaches to sample preparation frequently employ organic solvents, which are not only costly and potentially harmful to the environment but also pose health risks [7]. In response, the pursuit of automation in sample preparation has emerged as a highly effective strategy by streamlining the process and facilitating rapid, straightforward analytical methods [8]. It is worth noting that sample handling is a decisive factor in the productivity of an analytical program, with the phase alone consuming up to 80% of the total analysis time [9]. The quality of this step hinges on the merits of the method employed and the functionality of the analytical instrument [10]. Over recent decades, there has been a marked increase in efforts to refine and innovate sample preparation techniques, with a focus on preconcentration and separation.

Advanced materials, such as metal–organic frameworks (MOFs), have been harnessed to overcome the limitations of conventional materials and to diminish reliance on them. The development of several sorbent-based extraction methods has been a testament to this, striving to offer more efficient and environmentally benign alternatives to traditional solid-phase extraction [11]. Furthermore, deep eutectic solvent (DES) has garnered significant attention in recent years owing to its distinctive properties and potential applications across various fields [12]. One area of interest is the utilization of DESs in combination with MOFs to enhance their performance and functionality. Several studies have explored the use of DESs in conjunction with MOFs for different applications, ranging from adsorption of contaminants to catalytic reactions [13,14]. The advent of novel materials like MOFs and DESs has garnered considerable interest given their distinctive properties, which lend themselves well to sample preparation. This review delves into the confluence of DES and MOFs by examining the synergistic potential of their combination for the advancement of sample preparation methods. Specifically, this comprehensive review explores the application of DES-decorated MOFs in food and environmental sample preparation. We provide a detailed analysis of the characteristics of DES@MOF and its individual components, methods for decorating MOFs with DES, the advantages of these composite materials in sample pretreatment, and their specific applications in food safety and environmental monitoring. The review also discusses the current challenges and future directions of the composite, offering valuable insights for further research and development.

## 2. DES and MOFs

### 2.1. Basic Information About DES

DES exhibits chemical and physical properties akin to those of ionic liquids (ILs) [15]. Unlike ILs, the synthesis of DES boasts 100% atomic efficiency, it is solvent-free, and it does not necessitate additional purification, rendering it amenable to large-scale industrial applications [16]. The novel class of green solvents has emerged as a promising, environmentally benign alternative to traditional solvents [17,18]. DESs typically comprise two components at least, including a hydrogen bond donor (HBD) and a hydrogen bond acceptor (HBA), which exhibit a significantly reduced melting point compared to the pure components [19]. Additionally, atypical DESs can be governed by additional noncovalent interactions, including halogen bonding and chalcogen bonding [20]. They present several benefits, such as reduced toxicity, cost-effectiveness, biodegradability, and customizable properties. DESs are capable of dissolving a broad spectrum of compounds, positioning them as an ideal, eco-friendly, and non-hazardous substitute for conventional solvents [21]. The application of DES is currently expanding in the realm of green analytical chemistry [22] and the development of sensors and biosensors [23]. DES can be categorized into five distinct types based on their composition [24]: Type I comprises metal salts and quaternary ammonium salts; Type II consists of hydrated metal salts and quaternary ammonium salts [25]; Type III is characterized by the combination of HBDs and quaternary ammonium salts; Type IV involves hydrated metal salts and HBDs; and Type V is defined by the association of menthol/thymol analogues with HBDs. Over recent years, Type III has garnered considerable attention due to its enhanced selectivity in the extraction of diverse components and has been shown to exhibit reduced toxicity. In the Type III cocrystal system, exemplified by a mixture of urea and choline chloride (ChCl), urea functions as an HBD, while ChCl serves as an HBA. The intermolecular hydrogen bonding interactions within this system lead to a reduction in lattice energy, necessitating lower temperatures for melting and thus resulting in a decreased eutectic temperature [26].

**Figure 1:**
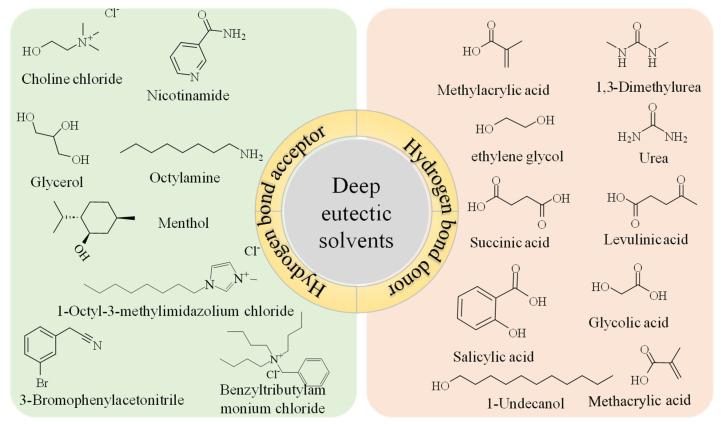
Structures of hydrogen bond donor and hydrogen bond acceptor of deep-eutectic-solvent-decorated metal–organic framework.

DESs are complex mixtures that encompass anions, cations, and neutral ligands. In contrast to ILs, which are composed exclusively of anionic and cationic species, and traditional molecular solvents that consist solely of neutral ligands, the varied composition of DESs confers upon them a high degree of adaptability for specific applications [27]. The structures and characteristics of the HBA and HBD directly affect the physicochemical properties of the resulting DESs. Consequently, by meticulously adjusting various aspects of HBA and HBD, a systematic design approach for DESs can be realized [28]. For instance, common HBAs, such as halogenated quaternary ammonium salts, can exhibit a range of hydrophilic to hydrophobic characteristics by modifying their carbon chain length. Additionally, the selection of different halogen atoms can introduce varying degrees of electronegativity within the DESs [29]. Furthermore, it is feasible to regulate the types of hydrogen bonding groups present in HBDs, including using exclusively carboxyl groups or combinations of carboxyl and amino groups, and it is feasible to adjust the quantity of hydrogen-bonding functional groups, for example, from a single to multiple carboxyl groups. Consequently, the manipulation of HBDs facilitates the creation of DESs with tailored hydrogen bonding capabilities [30]. Nonetheless, DESs also have certain limitations, and there are instances where it may be necessary to modify the intrinsic properties of DESs and simultaneously optimize additional factors to attain the highest extraction efficiency [31]. Structures of HBA and HBD of DES-decorated MOF are depicted in Figure 1. Some components of DES are inherently multifunctional, acting both as donors and acceptors in the formation of non-covalent bond networks [32,33,34].

### 2.2. MOFs and MOFs as Adsorbents

**Figure 2:**
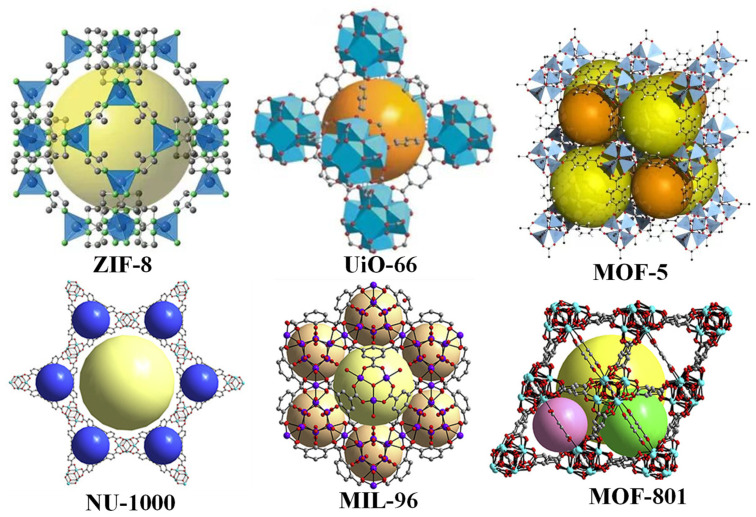
Structures of typical metal–organic frameworks.

MOFs represent a class of materials that have garnered significant attention due to their potential as solutions to a myriad of contemporary challenges across various sectors, including environmental management, industry, and healthcare. They address pressing issues, such as carbon capture, biomedical delivery, gas separation, chemical sensing, filtration, catalysis, and remediation, where their utility is substantial [35]. The high expectations for MOFs are directly associated with their remarkable characteristics, which include extensive surface areas, post-synthetic modifiability, pore selectivity, and customizability. MOFs are constructed akin to buildings by utilizing two primary components: (1) organic ligands that act as the pillars or the connectors of the structure, and (2) metal centers and clusters that serve as the anchor points or the nodes of these pillars [36]. Utilizing molecular building blocks, each MOF is meticulously assembled with a pore size, a distinct topology, and a pore environment. The synthesis of an MOF is conventionally achieved through solvothermal or hydrothermal methods, wherein crystals gradually form from a high-temperature solution. Figure 2 illustrates the structures of typical MOFs. Considering the diverse metal environments and the vast array of organic ligands, it is noteworthy that the number of reported MOFs reaches into the hundreds of thousands, with half a million more predicted. Approximately 100,000 MOFs have been documented in the Cambridge Structural Database [37].

Adsorption-based methods have demonstrated exceptional productivity for the enrichment and analysis of a diverse array of targets among the myriad of potential techniques. An effective adsorbent is characterized by its rapid kinetics, high adsorption capacity, and facile regeneration capabilities. In the current landscape, MOFs are being increasingly employed as sorbents for the preconcentration of different analytes owing to the multitude of interactions, such as size-selective adsorption, π–π interactions, dipole–dipole interactions, coordination interactions, hydrogen bonding, acid–base interactions, hydrophobic effects, and electrostatic interactions [38]. These interactions facilitate the adsorption process. MOFs play a crucial role in the analysis of various classes of targets, including drugs, pesticides, polycyclic aromatic hydrocarbons (PAHs), fungicides, nitro-aromatics, and persistent organic pollutants (POPs). The integration of electron-rich functional groups, such as amino (-NH_2_), can enhance the adsorptive capacity of MOFs toward specific analytes due to electrostatic interactions [39]. MOFs are efficiently utilized in a range of preconcentration techniques, including magnetic solid phase extraction, solid phase extraction, micro solid phase extraction, dispersive solid phase extraction, and so on [40]. The structure and properties of MOFs can be precisely tailored through the judicious selection of organic linkers and metal nodes, allowing for the creation of MOFs with target-specific adsorption properties. Their straightforward synthesis, extensive surface area, readily accessible dynamic functional groups, and stable system topology endow MOFs with an exceptional adsorption capacity for organic pollutants surpassing that of conventional adsorbents [41]. Furthermore, MOFs serve as the packing materials of the stationary phase in chromatographic columns, leveraging their extreme porosity, extensive surface topology, and exceptional thermal stability [42].

**Figure 3:**
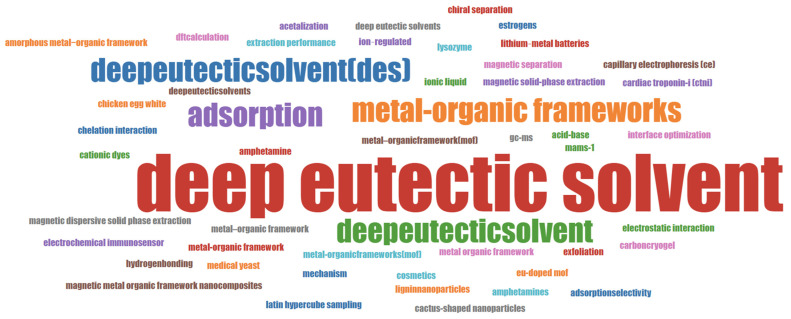
Word cloud map of the top keywords, with the font size indicating the frequency of occurrences.

Due to their adjustable nature for specific functions and robust chemical stability, MOFs hold significant promise in environmental purification [43]. They are particularly effective for harmful contaminant removal from water, and they offer a promising approach to mitigate the limitations related to electrode materials in supercapacitors [44]. Their potential for modification and structural adaptability present an opportunity to address these challenges [45]. The properties of MOFs can be customized by adjusting the synthesis parameters, including the selection of starting materials (organic ligands and metal precursors), control over synthesis conditions (ion concentration, pH, and temperature), and the use of various synthesis techniques (such as hydrothermal methods and microwave irradiation) [46]. Compared to traditional nanoporous materials, MOFs are particularly appealing due to their ability to integrate a variety of organic linkers and metals, resulting in a broad spectrum of materials with diverse crystal structures and chemical compositions. Their structural versatility and functionality make MOFs exceptional alternatives to traditional porous materials like zeolites. UiO-66-NH_2_, for instance, has demonstrated remarkable performance in the adsorption of dyes, heavy metals, and fatty aldehydes, attributed to its high surface area, thermal/chemical stability, and ease of external surface functionalization. Furthermore, their integration and cooperation with different functional materials to create composites have been shown to significantly enhance the performance of MOFs, as demonstrated in a recent study comparing the DES-modified MOF with the single MOF adsorbent for different sample pretreatments [47]. The word cloud in Figure 3 visually summarizes the most significant terms from the literature related to DES and MOFs, thus facilitating the identification of patterns, themes, or key terms. The prevalence of certain words, such as “deep eutectic solvent”, “metal-organic frameworks”, and “adsorption”, indicates that these two advanced materials hold significant potential in the fields of food sample analysis and environmental remediation.

## 3. The Role of DES in MOFs

### 3.1. Influence of DES on the MOF Structure

Characterized by an extensive surface area, tunable pore sizes, unsaturated coordination sites, high porosity, and permeability, MOFs have established themselves as innovative and promising materials [48,49]. They have been the subject of extensive research for their potential applications in drug delivery, gas storage, and environmental remediation [50]. MOFs are renowned for their advantageous properties, including tunable pore dimensions and an expansive surface area. However, a notable limitation of MOFs is their relatively poor chemical stability. By integrating MOFs into a diverse range of functional materials to create composite structures, a significant improvement in MOFs’ performance can be realized [16]. The application of DES in the synthesis of MOFs has recently attracted considerable attention [51]. DESs can alter the MOF’s structure by affecting the crystal morphology, regulating pore dimensions, bolstering the stability of the MOF, and so on. Liu et al. pioneered the ionothermal synthesis of Cu_3_(BTC)_2_ (BTC: benzene-1,3,5-tricarboxylic acid) by utilizing a DES blend of urea derivatives and ChCl. This method successfully yielded pure Cu_3_(BTC)_2_ crystals devoid of Cu_2_O and Cu impurities [52]. In a similar vein, Chen et al. detailed a multirooted synthesis of porous anionic frameworks leveraging DES, which displayed remarkable gas uptake capacity [53]. This finding underscores the potential of DES for augmenting the gas adsorption characteristics of MOFs. Zhang et al. elucidated the diverse structure-directing roles of DES in creating open metal sites and porosity for gas storage within MOFs, underscoring the pivotal role of DES in MOF preparation [54]. Moreover, Chen et al. synthesized a zinc(II)-boron(III)-imidazolate framework (ZBIF) with unique pentagonal channels using DES, exemplifying the broad utility of DES in MOF design [55]. Ma et al. also explored solvent-templated and chirality-controlled catenation isomerism in MOFs, indicating the influence of DES on the structural attributes of MOFs [56]. Their results demonstrated the potential of DES in influencing the properties of MOFs, opening up new possibilities for the development of chiral MOFs using environmentally friendly solvents. Li et al. showcased the selective CO_2_ capture capabilities of microporous 2D indium MOFs synthesized with DES, further underscoring the significance of DES in enhancing MOFs’ functionality [57]. A study conducted by Almanassra et al. elucidated the impact of reaction time on the characteristics of MOF-based nanoparticles [58]. The research effectively demonstrated that reaction time significantly affects porosity and particle size, which are pivotal for optimizing the performance of sorption mechanisms.

In recent studies, the synthesis of crystalline and amorphous MOFs with tailored properties for the adsorption of perfluoroalkyl substances (PFASs) has shown significant promise. Specifically, researchers have developed a green protocol utilizing DES to precisely control the particle size and introduce defects within crystalline and amorphous MOFs. This innovative method modulates the molar ratios of components, such as ammonium salts and glycolic acid or acetamide within the DES, to synthesize the proposed materials with varying degrees of structural disorder. The DES not only serves as a reaction medium but also plays a vital role in the modulation mechanism through the combined regulation of competitive coordination and deprotonation abilities. The resulting crystalline and amorphous UiO-66 exhibits larger pores and increased surface areas, which significantly enhance the accessibility of adsorption sites and facilitate the diffusion and adsorption of PFASs. This approach has led to the creation of MOFs with exceptional adsorption capacities, outperforming many traditional adsorbents and indicating the potential of DES-based synthesis for developing next-generation materials for environmental remediation [43,59].

### 3.2. DES’s Effect on the Performance of MOF

Research has predominantly focused on employing DESs as solvents for the MOFs’ synthesis. However, recent studies have endeavored to exploit the combined properties of these two classes of materials. DES-functionalized MOFs integrate the exceptional attributes of MOFs, such as porosity, a high surface area, and a diverse structure, with the solubilization capabilities of DESs. These composite materials demonstrate improved mechanical strength, stability, and guest uptake capacity relative to their unmodified counterparts, which are attributed to the synergistic effects between the DES and MOF constituents [60]. Homogeneous catalysts, while beneficial in many reactions, are not without drawbacks, including the potential for environmental contamination and challenges associated with product separation, which can hinder their application on an industrial scale [61]. Li and colleagues investigated an eco-friendly and highly effective catalytic system that integrates MOFs with DESs for the dehydration of fructose to yield 5-hydroxymethylfurfural (HMF) [62]. They assessed the impact of varying polyol quantities within the DESs on the reaction’s efficiency. Their research presents a broadly applicable approach for the creation of hybrid catalytic systems, thus facilitating the green synthesis of bio-based platform chemicals. Huynh et al. conducted a one-pot conversion of sugar to HMF and furfural, employing a MIL-101(Cr) as a highly active catalyst and a DES (lactic acid and ChCl) as a green solvent. This approach is an effective method for converting biomass into platform intermediates [63].

**Figure 4:**
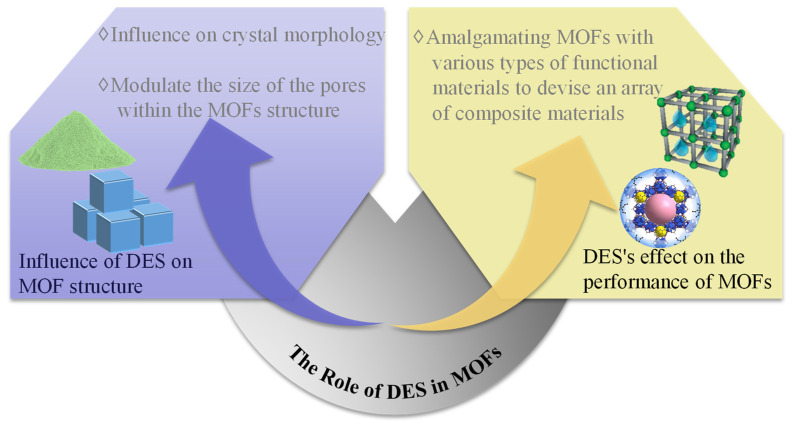
The role of deep eutectic solvent in a metal–organic framework.

The incorporation of DESs can also enhance the adsorption sites within MOFs or potentially introduce new functionalities, thereby facilitating the formation of noncovalent or electrostatic interactions that are pivotal to the extraction mechanism. In research by Wei et al. on DES-functionalized magnetic MOF composites for SPE of cationic dyes, it was discovered that the DES-modified adsorbent surface is rich in electronegative hydroxyl groups. These groups interact with cationic dyes through electrostatic forces, while hydrogen bonding between the nitrogen atoms of the dyes and the oxygen-containing functional groups of DES facilitates extraction. The DES-functionalized Fe_3_O_4_-NH_2_@HKUST-1, characterized by a high number of nitrogen atoms with lone pair electrons, was synthesized and combined with 3-acrylamidepropyltrimethylammonium chloride/D-sorbitol to form a functionalized amino magnetic MOF (HKUST-1) [64]. The roles of DES in MOFs are summarized in Figure 4.

## 4. DES-Decorated MOFs for Sample Preparation

### 4.1. Methodologies for Decorating MOFs with DES

The synthesis of MOFs can be tailored to achieve optimal performance for specific applications. Post-synthetic modification of MOFs allows for the customization of their structural properties, presenting significant opportunities for enhancing performance. Certain strategies, such as cation exchange, metal substitution, ligand exchange, and the incorporation of diverse functional metals and organic linkers, are well-established methods for tuning the porous structure of MOFs [65]. Furthermore, post-synthetic modification techniques are frequently employed. The integration of DESs into MOFs represents a novel approach for the creation of DES/MOF composites. The successful impregnation of DESs within MOFs is primarily attributed to the interactions between the DESs and the pore surfaces of the MOFs [66].

The wet impregnation method is the most commonly utilized post-synthetic technique for incorporating DESs into MOFs [67]. Initially, DESs are dissolved in a sufficient quantity of an inert solvent, such as acetone, methanol, or ethanol, to ensure optimal dispersion [68]. Subsequently, the MOF powder is introduced into the solution, which is then stirred at room temperature for several hours. Once a homogeneous mixture is achieved, the solvent is evaporated, yielding a powdered IL/MOF composite. Numerous studies have utilized this approach to fabricate DES/MOF composites for applications in adsorption, separation, and catalysis. Another frequently utilized post-synthetic modification method for MOFs is direct ligand functionalization. The selection of metal centers and organic ligands, including ions and clusters, is responsible for the structural and functional diversity of MOFs. The process of ligand functionalization has significantly increased the adaptability of these materials. Although ligand functionalization is capable of modifying the topology of MOFs, it typically does not alter the overall structure of the original compound. Conversely, it can substantially affect the porosity—encompassing the pore volume and the surface area—as well as the pore structure, including the pore shape and size. Most notably, it can significantly influence the surface functionality of the MOF pores. Direct ligand functionalization may involve the attachment of a functional reagent, such as a prepared DES, to the pre-synthesized MOFs, often modifying the ligands to expose free amino or carboxyl groups. Subsequently, acid–base condensation reactions are used to incorporate the DESs into the MOFs. To maintain the unaltered structure of a functionalized MOF, ligand functionalization should be conducted without impacting the primary functionality or the overall shape of the parent structure [69].

### 4.2. Extraction Technology Coupled with DES/MOF Composite Material

Common extraction methods coupled to DES/MOF composite material currently in use include solid-phase extraction (SPE), dispersive solid-phase extraction (DSPE), solid-phase microextraction (SPME), magnetic solid-phase extraction (MSPE), and molecularly imprinted solid-phase (micro) extraction (MISPE). The development of a more efficient sorbent is pivotal for advancing these techniques. The ongoing pursuit to ensure the stability of MOFs and to further enhance their performance is a frequently explored area. Utilizing appropriate DESs for the modification of MOFs can effectively mitigate the impact of MOFs’ inherent deficiencies on sample preparation [70]. Table 1 illustrates the recent applications of DES/MOF composites for sample preparation [4,7,21,30,47,64,71,72,73,74,75,76,77,78,79,80,81,82].

#### 4.2.1. Solid-Phase Extraction

**Figure 5:**
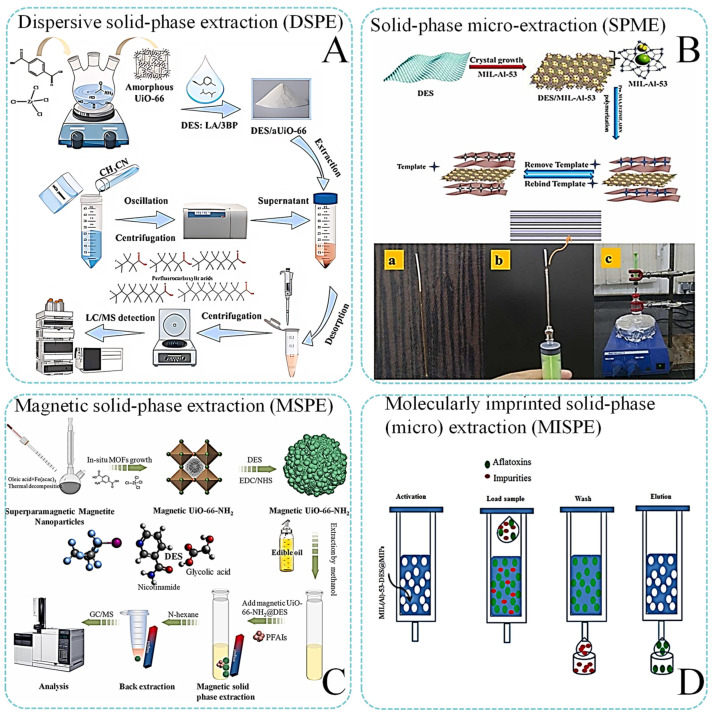
Extraction technology coupled to deep eutectic solvent/metal–organic framework composite material. (**A**) Dispersive solid-phase extraction (DSPE) [47]. (**B**) Solid-phase microextraction (SPME) [78]: (a) Single fiber in mold; (b) fiber bundle; (c) SPME setup. (**C**) Magnetic solid-phase extraction (MSPE) [73]. (**D**) Molecularly imprinted solid-phase (micro) extraction (MISPE) [4]. Reprinted with permission from refs. [4,47,73,78], Elsevier Publishing.

SPE is a chromatography-based sample pretreatment technique that leverages selective adsorption and elution to concentrate, separate, and purify samples (Figure 5A). This method is recognized for its moderate selectivity, rapid separation, substantial recovery, and the ability to prevent emulsification compared to conventional liquid–liquid extraction [83]. With the advancement of this technology, numerous related sample pretreatment techniques have evolved. SPE-based technologies have gained increasing favor over alternative methods due to their diverse range of adsorbents that cater to various polarities and functionalities. These technologies are also noted for their high potential for automation, enhanced efficiency, and user-friendly operation [59]. The choice of extraction solvent is also a critical factor in the efficacy of SPE procedures [64,84]. Ozalp et al. synthesized and characterized a MOF-DES nanocomposite material. The NH_2_-MIL-53(Al)-DES (ChCl-Urea) nanocomposite, utilized as a solid-phase adsorbent, was effective in extracting Rhodamine 6G from aqueous and cosmetic matrices. The material demonstrated its advantages through optimal dosage (15 mg), a short adsorption time (3 min), recyclability (15 cycles), and high recovery ranging from 95.0% to 102.0%. The proposed SPE/ultraviolet-visible spectrophotometry method, which is sensitive, practical, precise, and cost-effective, was validated for dye analysis, proving suitable for this application [81]. In another study, Ozalp et al. prepared MIL-101(Cr)@DES (ChCl-urea) nanocomposites via the impregnation method for use as a solid-phase adsorbent in the extraction of imidacloprid from herbal tea and water samples, followed by high-performance liquid chromatography–ultraviolet detection (HPLC-UV) analysis [71]. The optimal SPE conditions included an adsorbent mass of 10.0 mg, a pH of 7.0, an adsorption time of 3 min, and an elution volume of 2.0 mL, yielding recoveries between 90% and 98% for both tea and water samples. With a sample volume of 40 mL, the method provided an enrichment factor of 20. It possessed excellent sensitivity, adsorption capacity, speed, recovery, and reuse potential, making it well-suited for herbal tea and water sample analysis. The SPE-HPLC method’s recovery confirmed that the nanocomposite material offers high extraction efficiency for the removal of imidacloprid from herbal tea and water samples. This composite material presents significant benefits regarding cost, environmental impact, and time efficiency.

#### 4.2.2. Solid-Phase Microextraction

SPME is a straightforward, efficient, and solventless sample preparation technique that has found broad application in environmental, food, and forensic analyses [84]. The objective of SPME is to attain swift equilibrium between the analytes within the sample matrix and the SPME device’s coating [85]. This technique employs a fused silica fiber coated with an extracting phase, typically a polymer, which can be directly immersed in the sample or exposed via the headspace (Figure 5B). Analytes are absorbed by the coating and subsequently thermally desorbed for gas chromatography (GC) analysis or solvent-rinsed for HPLC analysis. SPME significantly minimizes the sample pretreatment time, as it combines extraction, injection, and concentration steps [86]. Ezel Boyac et al. developed a high-throughput, fully automated method that integrates thin-film solid-phase microextraction (TF-SPME) with liquid chromatography–mass spectrometry (LC-MS/MS) for the simultaneous quantitative analysis of 110 doping compounds. The SPME procedure was optimized with respect to the extraction pH, the solution’s ionic strength, the washing solution, and the durations of extraction and desorption, specifically for urine sample analysis. This method is capable of extracting not only free compounds but also glucuronidated forms of analytes. The proposed assay facilitates reliable and rapid analysis of a multitude of prohibited substances, presenting an appealing alternative to conventional methodologies utilized in anti-doping laboratories [87].

Jafari et al. synthesized hybrid MOFs with high specific surface areas, consisting of ZIF-90 and ZIF-8, which were deposited on graphene oxide (GO) to create novel adsorbents. These ZIF-8-90@GO hybrids were integrated vertically within the pores of hollow fibers (HFs) for the extraction of three types of phthalates using a hollow fiber–solid phase microextraction–deep eutectic solvents–high-performance liquid chromatography–ultraviolet (HF-SPME-DES-HPLC-UV) device. The utilization of DES for the back extraction step contributed to the green chemistry approach of the method. The influential parameters in the extraction and desorption processes were meticulously optimized to ensure maximum efficiency and sensitivity. The optimized method exhibited linearity in the range of 0.1 to 500 µg/L, with detection limits (LODs) between 0.026 and 0.058 µg/L, quantification limits (LOQs) ranging from 0.089 to 0.194 µg/L, relative standard deviations (RSDs%) of 1.8 to 4.7%, and enrichment factors (EFs) of 45.4 to 47.1. The extraction recoveries for dimethyl phthalate (DMP), dibutyl phthalate (DBP), and diethyl phthalate (DEP) were 90%, 91%, and 87%, respectively. The relative recoveries for the real samples were in the range of 90–103%. The presence of methyl and aldehyde groups in the synthesized hybrid adsorbents enhanced both hydrophilic and hydrophobic interactions, thereby improving extraction efficiency, especially with an increasing number of study species. This methodology is capable of effectively extracting analytes that exhibit strong interactions with the prepared adsorbent. Consequently, the method has potential applications for the extraction of other targets and the analysis of various vegetable and fruit samples [72].

#### 4.2.3. Dispersive Solid-Phase Extraction and Magnetic Solid-Phase Extraction

DSPE has arisen as an alternative method to traditional SPE (Figure 5C). Although SPE is among the most prevalent techniques for sample preparation, it necessitates the use of disposable cartridges and substantial elution solvents and is frequently time-consuming. The expedited sample preparation times and cost-effectiveness associated with DSPE have contributed to its increasing popularity. In this method, the solid sorbent is directly dispersed into the sample matrix. Following the adsorption of analytes, the sorbent is retrieved, and the target compounds are subsequently eluted. Several principal sorbent separation techniques are employed in DSPE: filtration, centrifugation, and magnetic collection. At present, MSPE represents the predominant variant of DSPE, with DESs either applied to the surfaces of solid sorbents or utilized to create a magnetic fluid [88]. It is an advanced extraction technique that employs magnetic particles as an adsorbent [77]. The primary advantage of MSPE is its highly efficient and rapid separation process, which allows for the straightforward isolation of magnetic particle-bound compounds from the sample matrix using a magnetic field. MSPE is extensively applied in environmental and biological sample analyses owing to its convenience and superior efficiency. This method capitalizes on the magnetic properties of the extractant and its specific affinity for target analytes, enabling the swift enrichment of these targets. Moreover, MSPE minimizes the usage of organic solvents and simplifies the extraction procedure. It also facilitates the recovery and reuse of materials, establishing itself as an eco-friendly, straightforward, and promising technology for sample pretreatment. The exploration and development of innovative materials for MSPE are crucial for achieving effective separation and concentration of substances of interest [89]. A study focused on the preparation of nanocomposites for extracting estrogens in cosmetics, showcasing the versatility of DES-MOF composites in analytical chemistry applications [79].

In a remarkable advancement for food safety monitoring, Fan et al. [47] have consistently dedicated their efforts to this research field. They developed an innovative method using DES-functionalized amorphous UiO-66 (DES/aUiO-66) as an adsorbent for the DSPE of perfluoroalkyl substances (PFASs) from infant milk powder. This method stands out for its simplicity, speed, and ultrasensitive detection capabilities with a detection limit as low as 0.330–0.529 ng/kg. The researchers utilized advanced computational techniques, such as ab initio molecular dynamics (AIMD) simulations, to elucidate the mechanism behind the material’s high adsorption efficiency. The method’s validation against real-world samples confirmed its practicality and potential to significantly enhance the analysis of trace PFASs in milk powder, contributing to a safer food supply for infants. This pioneering work encapsulates the convergence of green chemistry, advanced materials, and cutting-edge analytical techniques to address pressing food safety challenges. In 2023, they conducted research on the synthesis of a novel DES functionalized magnetic MOF, which was utilized as an adsorbent for the extraction of perfluoroalkyl iodides (PFAIs) from edible oils. A sensitive extraction method was developed employing MSPE based on UiO-66-NH_2_@DES in conjunction with gas chromatography–mass spectrometry (GC-MS). The study investigated the impact of methanol extraction duration, various adsorbents, adsorbent mass, and MSPE extraction time to optimize extraction efficiency. The magnetic UiO-66-NH_2_@nicotinamide-acetic acid system (with an HBA/HBD molar ratio of 4:1, 50 mg, and 10 min) was ultimately selected for the enrichment of PFAIs from a 5 mL methanol extract, followed by elution with n-hexane. Under the optimized conditions, the method demonstrated enhanced sensitivity, favorable recovery rates, and a detection limit ranging from 2.81 to 34.3 pg/g. The method was effectively applied to analyze PFAIs in multiple edible oils, detecting target compounds at concentrations between 212.9 and 3053 pg/g. This research underscores the importance of targeted design in the development of function-oriented materials for trace food contaminant analysis, offering new insights into the advancement of PFAS sample preparation methods [73]. Then, a DES functionalized magnetic MOF adsorbent was developed for the analysis of fluorotelomer alcohols (FTOHs) in edible oils, addressing significant environmental and food safety concerns. The innovative adsorbent, synthesized by immobilizing a levulinic acid/trifluoromethoxybenzene-based DES onto magnetic UiO-66-NH_2_, demonstrated exceptional specificity and sensitivity for FTOHs, with a detection limit ranging from 31.51 to 185.76 pg/g and satisfactory recoveries between 73.83% and 128.55%. The method’s effectiveness was validated through the analysis of 12 different edible oil samples, revealing the presence of FTOHs at concentrations varying from 401.89 to 8006.18 pg/g. This research not only introduced a green and efficient approach for the enrichment and detection of emerging food contaminants but also provided critical insights into the underlying mechanisms of interactions between DES and FTOHs through comprehensive quantum chemical calculations, ^19^F NMR, and ^1^H NMR. The study represents a significant advancement in the application of MOF-based materials for food safety monitoring, suggesting the potential of DES-functionalized MOFs in analytical chemistry [82].

Liu et al. introduced an innovative MSPE technique for the extraction of pyrethroid pesticides from environmental water samples, followed by determination using gas chromatography coupled with tandem triple quadrupole mass spectrometry. In their study, they prepared an adsorbent, denoted as M-ZIF-8@DES, by coating the surface of M-ZIF-8 with a DES. Material characterization revealed that M-ZIF-8@DES possesses favorable magnetic properties (61.3 emu/g), substantial pore volume (0.292 mL/g), and an expansive specific surface area (96.83 m^2^/g). Single-factor experiments were conducted to assess the influence of different parameters on the MSPE’s performance. The developed method was successfully applied to the determination of pyrethroid pesticides in environmental water samples. This research underscores the potential of DES-modified MOF in a variety of sample pretreatment techniques [90]. Meng et al. presented a study on the fabrication of a cactus-shaped magnetic composite, specifically UiO-66-NH_2_, for SPE of RNA. This composite comprises the UiO-66-NH_2_, which has been functionalized with Fe_3_O_4_ nanoparticles. The composite was then dispersed in a lactic-acid-based DES, resulting in Fe_3_O_4_-COOH@UiO-66-NH_2_@DES. The structure of the sorbents was meticulously characterized through a suite of analytical techniques. The synthesis of this innovative material has led to the optimization of the sorbents’ extraction performance, reaching a maximum adsorption capacity of 246 mg/g. The sorbent demonstrated the ability to selectively extract RNA from a mixture containing bovine hemoglobin, DNA, and amino acids, and it could be efficiently separated and recovered using a magnetic field for RNA SPE. Studies on sorbent regeneration revealed that it could be reused multiple times without significant loss of extraction capacity following regeneration with DES. The successful extraction of RNA from yeast confirmed the practical utility of this sorbent. This research introduces a novel method for the separation and enrichment of RNA [21]. Wei et al. synthesized an array of seven DESs formulated from quaternary ammonium salts and either acetic or lactic acid. These DESs were strategically employed to modulate a magnetic MOF composite, namely Fe_3_O_4_-UiO-66-NH_2_ (MUiO-66-NH_2_). Through precise control over the DES structure, they achieved specific regulation of the UiO-66-NH_2′_s properties. The DES-MUiO-66-NH_2_ adsorbent combines the extensive specific surface area and reactive sites of MOFs with the diverse reactive functional groups of the DESs. Post-modification with benzyltributylammonium chloride-based DES, the composite’s morphology underwent a substantial transformation, with spherical particles becoming coated by an irregular layer, leading to enhanced surface roughness. The uniform surface of the DES-MUiO-66-NH_2_ adsorbent facilitated the chemical adsorption of personal care and pharmaceutical products (PPCPs), notably improving the adsorbent’s capacity for ofloxacin and ibuprofen. The DES-MUiO-66-NH_2_ demonstrated an enhanced ability to capture PPCPs through a variety of interactions, marking a significant advancement in adsorption capacity and selectivity [76]. The study highlighted the guiding role of DES in selective adsorption, indicating the potential of this approach for efficient removal of PPCPs from water systems.

#### 4.2.4. Molecularly Imprinted Solid-Phase (Micro) Extraction

MISPE is an exceptionally selective variant of SPE that leverages polymers imprinted with the molecular structure of a target analyte or a class of compounds (Figure 5D). The imprinting process is initiated by blending a template molecule with functional and cross-linking monomers, followed by polymerization and subsequent removal of the template. This procedure leaves behind specific binding sites within the polymer matrix, thereby conferring a high degree of selectivity for the target molecules, which in turn diminishes interferences and augments extraction efficiency [91]. The characteristics of MOFs can be markedly enhanced through the utilization of molecularly imprinted polymers (MIPs) in the preparation of compounds. This approach employs DES as the functional monomer, further refining the properties of the resultant MOFs. In a pioneering study, Fatemeh and colleagues synthesized an immunoaffinity column under the designation MIL(Al)-53-DES@MIPs [2]. The proposed synthesis strategy led to a marked expansion in the surface area and the availability of active sites for analyte adsorption, consequently amplifying the column’s performance and its efficacy in capturing target analytes. Moreover, the use of MIPs in combination with MOFs and DESs has been investigated for the determination of antibiotics in meat and dairy products [92]. The study demonstrated the potential of DES@UMCM-1MOF/MIP for efficient extraction and analysis of antibiotics, highlighting the synergistic effects of combining these materials.

Cheng et al. developed a surface-imprinted polymer (DES-MMIP) that exhibits superior separation and adsorption characteristics. This polymer is based on magnetic UiO-66-NH_2_ as a carrier and utilizes DES as a functional monomer, and it is designed for the rapid and selective extraction of benzedrine hydrochloride (BDH) from environmental samples. The study comprehensively investigated the structure and properties of DES-MMIP, optimizing the factors that influence equilibrium experiments. The experimental findings indicate that DES-MMIP possesses a maximum adsorption capacity of 41.18 mg/g for BDH, achieving a rapid adsorption equilibrium within 30 min. The inclusion of magnetic UiO-66-NH_2_ and DES endows DES-MMIP with excellent separability, and its adsorption capacity for BDH is 1.4 to 2.6 times higher than that of other commercial adsorbents, such as hydrophile–lipophile balance material, zeolite, and silica gel. Moreover, DES-MMIP can be reused up to five times, maintaining a satisfactory recovery rate. These research outcomes offer a novel and promising approach for the fabrication of efficient adsorbents aimed at the swift and selective removal of BDH from environmental sources. The magnetic UiO-66-NH_2_, serving as a carrier, also holds potential for the development of other highly efficient adsorbents [7]. Han et al. conducted research focused on the selective recognition and adsorption of bovine hemoglobin (BHb), employing DES as functional monomers and BHb as template molecules. They synthesized a novel imprinted polymer based on MOFs using a surface polymerization technique. The MOF served as a substrate to enhance the accessibility of the imprinting sites, while DES was used as a functional monomer to generate a variety of forces on BHb, facilitating the creation of imprinting sites. The imprinted polymer film provided selectivity for the analyte. The study characterized and evaluated the prepared MOF@DES-MIPs and examined the impact of BHb concentration and adsorption time on the performance of MOF@DES-MIPs. It was observed that BHb adsorption onto MOF@DES-MIPs adheres to a monolayer adsorption model, with a maximum adsorption capacity of 151.28 mg/g. MOF@DES-MIPs displayed a high adsorption capacity and rapid mass transfer capability for the template protein BHb. This strategic approach established by the study sets a foundation for the selective recognition and rapid adsorption of BHb from complex matrices, offering promising prospects for the development of adsorbents for other proteins [74].

In an effort to address the primary issues of poor chemical stability and instability in the chemical environments of MOFs, for the first time, hollow and monolithic fibers based on metal–organic framework–deep eutectic solvents/molecularly imprinted polymers (MOF-DES/MIPs) were prepared by Mirzajani et al. Hollow fiber liquid membrane-protected solid-phase microextraction (HFLMP-SPME) coupled with gas chromatography–flame ionization detection methods were employed for the microextraction of phthalates. Several parameters impacting the extraction recoveries of phthalates were studied and optimized, including adsorption and desorption conditions. Under optimal conditions, the limit of detection (S/N = 3) of the method ranged from 0.008 to 0.032 µg/L, which is significantly lower than previously reported methods, with a quantification limit (S/N = 10) of 0.028 to 0.12 µg/L. Inter-day and intra-day precisions of this method, expressed as the relative standard deviation (RSD), were within 2.6 to 3.4% and 2.4 to 4.7%, respectively. The method was successfully applied for the determination of phthalates in 24 soybean oil samples, water, and yogurt, yielding satisfactory results [80]. Zhao et al. addressed the limitations associated with protein template molecularly imprinted polymers, such as their large molecular size, poor mass transfer properties, slow recognition kinetics, complex structure and conformation, and challenges with regard to separation and purification. The resulting UiO-66@DES-MIPs exhibited notable adsorption characteristics and selectivity. After six cycles of adsorption–elution, the adsorption loss of UiO-66@DES-MIPs was a mere 8%, indicative of their excellent repeatability and stability [75].

## 5. Others

The integration of different advanced sample preparation techniques has been a focal point in the field of chemical analysis, particularly for the detection of trace contaminants in complex matrices. This approach is exemplified by recent studies that combine modern extraction techniques, such as DSPE and DES-based dispersive liquid–liquid microextraction (DES-DLLME). In these studies, DES is not used as a modifier on the surface of MOFs but rather serves as a microextraction solvent or eluent [93,94,95,96,97,98]. DSPE, as highlighted in a study by Yu et al., involves blending a solid sample with a sorbent, followed by elution and analysis [97]. This method was significantly advanced by incorporating magnetic ZIF-8 as a sorbent and dispersant, thus leveraging its magnetic properties for easy recovery. The use of hydrophilic DESs as green alternatives to traditional organic solvents further enhanced the method’s environmental sustainability. The optimized method demonstrated low LODs and LOQs, with good linearity, precision, and recovery rates, and it is suitable for the analysis of organic pollutants in solid samples. Another study by Daghi et al. focused on the extraction of pesticides from fruit juices using an MOF-based DSPE combined with DES-DLLME [93]. The MOF, MIL–88A, served as an efficient sorbent, and the elution with water-miscible DES improved the extraction recoveries. The method offered high enrichment factors, wide linear ranges, and low LODs and LOQs, illustrating its potential for sensitive and reliable pesticide residue analysis in fruit juices. In addition, a study by Jouyban et al. introduced a novel solid-phase extraction approach termed homogenous dispersive solid-phase extraction (HDSPE) for the extraction of hydroxylated metabolites of polycyclic aromatic hydrocarbons (PAHs) from urine samples [96]. The method employed polyvinylpyrrolidone (PVP) as a sorbent and a DES as an elution solvent prior to instrumental analysis. The optimized method exhibited low LODs, broad linear ranges, and good precision, making it suitable for the determination of PAH metabolites in biological samples.

## 6. Evaluation of the Greenness Profile of the Developed Methods Using DES@MOF

**Figure 6:**
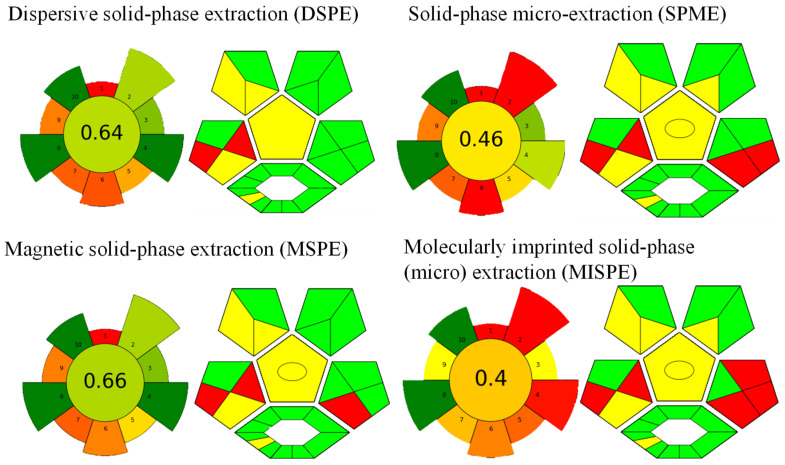
AGREEprep and ComplexMoGAPI scores of four methods mentioned in Figure 5.

Pena-Pereira et al. introduced the AGREE tool, designed to assess the environmental sustainability of analytical methods in accordance with 12 green analytical chemistry principles [99]. Subsequently, Wojnowski et al. enhanced the AGREE tool, developing the analytical greenness metric for sample preparation (AGREEprep) in 2022 [100]. This updated tool adheres to contemporary green sample preparation principles, highlighting the critical role of sample preparation in analytical processes. AGREEprep consists of 10 principles, each recalibrated with an effect score ranging from 0 to 1, which indicates the degree of impact associated with fulfilling each criterion. The subpart’s color gradient shifts from green to red, corresponding to the entered values and conditions. In 2021, an adjunct tool named the complementary green analytical procedure index (ComplexGAPI) was introduced to augment the evaluation process of sustainability in analytical procedures [101]. ComplexGAPI integrates an additional hexagonal segment into the original GAPI diagram, signifying the activities conducted prior to sample preparation and subsequent analysis. This supplementary hexagon facilitates the assessment of sustainability across a spectrum of parameters, encompassing production yields and conditions, selection of reagents and solvents, application of instrumentation and techniques, and purification methodologies. These criteria are pertinent for appraising the environmental friendliness of processes within the solvents, nanomaterials, stationary phases, and synthesis of organic compounds [102]. Figure 6 illustrates the AGREEprep and ComplexMoGAPI scores of four methods mentioned in Figure 5. A direct comparison between the results shows that both the SPME and MISPE procedures were relatively less environmentally friendly among the methods assessed here. Enhancements are needed for the sample throughput organic solvent consumption and manual mode, while the choice of the post-sample preparation configuration for analysis significantly impacts the scores. Nonetheless, these processes are largely dependent on the type of sample, whether it is a solid, liquid, or complex matrix sample.

## 7. Conclusions and Future Perspectives

DES and MOFs are distinguished by their unique attributes that surpass those of other materials in several domains, including catalysis, adsorption, sensing, and energy storage [31]. DES, known for its exceptional solvent characteristics and chemical stability, serves as both a stabilizer and an enhancer within MOF structures. This dual role improves the thermal stability and hydrophobicity of MOFs, thus expanding their range of applications. Preliminary research has unveiled the substantial potential of the DES@MOF composite, garnering escalating scholarly interest in the synthesis and application of these composites across various fields. Although approximately dozens of scholarly articles related to DES@MOF are indexed in literature databases—a notable increase from previous years—the research on DES@MOF is still relatively scarce compared to other analogous materials. Presently, DES@MOF research is in its nascent phase, predominantly centered on synthesis and performance evaluation. By manipulating the components of DES and MOFs, researchers have investigated diverse DES@MOF composites, yielding a spectrum of distinct properties. The DES modification endows DES@MOF with augmented stability, conductivity, and adjustability compared to unmodified MOFs. Certain DESs can further augment the environmental friendliness and cost-effectiveness of the resultant DES@MOF composites.

The practical application of DES@MOF in real-world scenarios presents formidable challenges. Identifying and producing the optimal DES@MOF composite is a technologically sophisticated endeavor with inherent complexity, necessitating further research on the compound’s stability and performance under diverse conditions. The majority of current research on DES@MOF performance is predicated on small-scale laboratory tests, and scaling up production while preserving the initial performance presents a significant hurdle. While the incorporation of specific DESs can make the composite eco-friendlier and more economical, the multitude of DES and MOF types, along with their sources, the intricacy of processing, potential material scarcity, and elevated costs, may impede large-scale manufacturing. Additionally, the potential for creating harmful byproducts during production, as well as concerns regarding recycling and post-use disposal, warrant more profound scrutiny. Addressing these challenges will be crucial for future research endeavors. It is through persistent exploration and resolution of these issues that the full potential of DES@MOF applications can be unearthed.

Furthermore, a deeper understanding of the interaction mechanisms between DES, MOFs, and target analyte is crucial for further development of these materials. Advanced characterization techniques and molecular modeling studies can provide valuable insights. Developing multifunctional DES-MOF composites (such as stimuli-responsive materials) that can perform multiple tasks simultaneously is an exciting direction for future research. Integrating DES-MOF composite materials with advanced analytical techniques can further enhance their application potential, such as developing online coupling systems with chromatographic or spectroscopic techniques and incorporating these materials into microfluidic devices for miniaturized analysis. DES-decorated MOFs represent a promising class of materials for food and environmental sample preparation. Their unique properties, combining the advantages of both DES and MOFs, offer significant improvements in extraction efficiency, selectivity, and environmental compatibility compared to traditional methods. With considerable developmental prospects in certain areas, such as catalysis, gas adsorption and storage, pollutant elimination, drug delivery systems, sensing materials, and battery components, the integration of DES within MOFs could lead to the creation of highly porous, customizable, versatile, and multifunctional materials with enhanced performance. The DES@MOF composite’s superiority across various sectors is anticipated to be substantiated through more profound and exhaustive research in the forthcoming years.

## Figures and Tables

**Table 1:** Application of deep eutectic solvent/metal–organic framework composite for sample preparation.

Adsorbent	Analytes	Matrix	Sample Preparation Method	Analytical Instrumentation	LOD (μg/L)	Amount of Adsorbent (mg)	Elution Volume (mL)	EF	Recovery (%)	Ref.
Fe_3_O_4_-COOH@UiO-66-NH_2_@DES (Tetrabutylammonium chloride: lactic acid)	RNA	DNA, bovine hemoglobin, and amino acids	MSPE	UV-Vis spectrophotometer	2.8	5	1	246	90~97	[21]
DES-magnetic UiO-66-NH_2_ MMIP (ChCl: ɑ-methylacrylic acid)	BDH	Environmental samples	MISPE	UV-Vis spectrophotometer	—	26.52	10	41.18	More than 90	[7]
NH_2_-MIL-53(Al)-DES (ChCl: Urea)	Rh 6G	Water and cosmetic sample	SPE	UV-Vis spectrophotometer	9.80	15	2	20	95.0~102.0	[81]
MIL-101 (Cr)@DES (ChCl-Urea 1:2)	Imidacloprid	Traditional herbal tea and water	SPE	HPLC-UV	0.144	10	2	20	90~98	[71]
DES-based ZIF-8-90@GO (ChCl: ethylene glycol)	Phthalates	Fruit and vegetable	SPME	HPLC-UV	0.026–0.058	—	0.2	45.4~47.1	90~103	[72]
Magnetic UiO-66-NH_2_@DES system (Nicotinamide: glycolic acid)	Perfluoroalkyl iodides	Edible oils	MSPE	GC-MS	2.81–34.3	5	2	—	74.9~111	[73]
MOF-199@DES-MIPs (ChCl: methacrylic acid)	BHb	Real bovine blood	MISPE	UV spectroscopy	—	151.28	20	150	78~82	[74]
UiO-66@DES-MIPs (ChCl: 1,3-dimethylurea)	Lyz	Egg white	MISPE	UV spectroscopy	—	243.87 ± 4.88	10	10–50	92	[75]
UMCM-1-DES/MIPs (Glycerol: quaternary ammonium chloride)	Phthalates	Yogurt and water, as well as in soybean oil	SPME	GC-FID	0.008–0.0320	6	7	441–446	95.5~99	[80]
DES-MUiO-66-NH_2_ (Benzyltributylammonium chloride: glycolic acid)	PPCPs	Water	SPE	UV-Vis spectrophotometer	1.6	5	1	—	More than 80	[76]
DES/Zn-MOF (Octylamine: succinic acid)	Carmoisine	Water and food samples	MSPE	UV-Vis spectrophotometer	2.4	10	1	34.38	92.3~99.8	[77]
MIL-Al (53)-DES/MIP (ChCl: glycerol)	Amphetamines and modafinil	Unauthorized medicinal supplements	SPME	GC-MS	0.023–0.033	1.6	0.15	163	95.14~104.63	[78]
DES/aUiO-66 (3-Bromophenylacetonitrile: levulinic acid)	Perfluoroalkyl carboxylic acids	Infant milk powders	DSPE	LC-MS	0.330–0.529	10	0.3	—	69~118	[47]
MGO@ZIF-8@DES (1-Octyl-3-methylimidazolium chloride: 1-undecanol)	ETD, ETO, DST	Cosmetics (toner, lotion, and cream)	MSPE	HPLC-UV	0.02–0.03	16	5	—	83.5~95.9	[79]
MIL(Al)-53-DES@MIPs (Quaternary ammonium chloride: glycerol)	Aflatoxins	Cereals	SPE	HPLC-FLD	0.023- 0.033	1.6	5	158.7–163.0	95.3~98.5	[4]
Fe_3_O_4_@MIL-101(Cr)@DES (Menthol: lactic acid)	NSAID	Lake and river water samples	MSPE	HPLC-UV	0.20–1.1	15	40	50–105	83.8~110.4	[30]
Fe_3_O_4_-NH_2_@HKUST-1@DES (APTMACl: D-sorbitol)	Cationic dyes	Fish samples	MSPE	UV-vis spectrophotometer	—	5	4	—	95.29~98.03	[64]
Magnetic UiO-66-NH_2_@DES (Levulinic acid: trifluoromethoxybenzene)	Fluorotelomer alcohols	Edible oils	MSPE	GC-MS	31.51–185.76	2	5	31.51–185.76	73.83~128.55	[82]

FLD: fluorescence detection. GC-FID: gas chromatography–flame-ionization detector. GC-MS: gas chromatography–mass spectrometry. MS: mass spectrometry. HPLC-UV: high-performance liquid chromatography–ultraviolet detection. UV-Vis spectrophotometer: ultraviolet-visible spectrophotometer. ChCl: choline chloride. MISPE: molecularly imprinted solid-phase (micro) extraction. MSPE: magnetic solid-phase extraction. SPME: solid-phase microextraction. SPE: solid-phase extraction. DSPE: dispersive solid-phase extraction. BDH: benzydamine hydrochloride. BHb: bovine hemoglobin. Lyz: lysozyme. LOD: limit of detection. EF: enrichment factor. PPCPs: pharmaceuticals and personal care products. ETD: estradiol. ETO: estrone. DST: diethylstilbestrol. APTMACl: 3-Acrylamidopropyl trimethylammonium chloride. NSAID: non-steroidal anti-inflammatory drugs; —: unmentioned.

## Data Availability

The original contributions presented in the study are included in the article, further inquiries can be directed to the corresponding author.
